# Silica-Based Nanoparticle Uptake and Cellular Response by Primary Microglia

**DOI:** 10.1289/ehp.0901534

**Published:** 2009-12-21

**Authors:** Judy Choi, Qingdong Zheng, Howard E. Katz, Tomás R. Guilarte

**Affiliations:** 1 Department of Environmental Health Sciences, Johns Hopkins Bloomberg School of Public Health, Baltimore, Maryland, USA; 2 Department of Materials Science and Engineering, Johns Hopkins University, Baltimore, Maryland, USA

**Keywords:** inflammation, microglia, nanoparticle, neurotoxicity, ROS, RNS, silica

## Abstract

**Background:**

Silica nanoparticles (SiNPs) are being formulated for cellular imaging and for nonviral gene delivery in the central nervous system (CNS), but it is unclear what potential effects SiNPs can elicit once they enter the CNS. As the resident macrophages of the CNS, microglia are the cells most likely to respond to SiNP entry into the brain. Upon activation, they are capable of undergoing morphological and functional changes.

**Objective:**

We examined the effects of SiNP exposure using primary rat microglia.

**Methods:**

We observed microglial uptake of SiNPs using transmission electron and fluorescence confocal microscopy. Microglial functions, including phagocytosis, generation of reactive oxygen species (ROS) and reactive nitrogen species (RNS), expression of proinflammatory genes, and cytokine release, were measured after SiNP exposure at different concentrations.

**Results:**

Microglia are capable of avidly taking up SiNPs at all concentrations tested. These same concentrations did not elicit cytotoxicity or a change in phagocytic activity. SiNPs did increase the productions of both intracellular ROS and RNS. We also observed a significant decrease in tumor necrosis factor-α gene expression at all concentrations tested and a significant increase in *COX-2* (cyclooxygenase-2) gene expression at the highest concentration of SiNPs. Analysis of cytokine release showed a detectable level of interleukin-1β.

**Conclusions:**

This is the first study demonstrating the *in vitro* effects of SiNPs in primary microglia. Our findings suggest that very low levels of SiNPs are capable of altering microglial function. Increased ROS and RNS production, changes in proinflammatory genes, and cytokine release may not only adversely affect microglial function but also affect surrounding neurons.

The use of engineered nanoparticles (NPs) has increased significantly in the past decade. Because of the flexibility of producing NPs of different sizes, shapes, and surface modifications, engineered NPs can be used in a variety of applications, including skin care products, foods, electronics, and medicine. Anthropogenic sources of NPs also emerge from power plants and industries. It is likely that humans will be exposed to NPs through dermal absorption, ingestion, and inhalation. This may pose a health concern because studies have shown that NPs can elicit adverse cellular effects *in vitro* (Fubini and Hubbard 2003; Napierska et al. 2009; Park and Park 2009). A recent study has shown that workers occupationally exposed to NPs for 5–13 months exhibited symptoms of pulmonary fibrosis, with NPs present in the cytoplasm of pulmonary epithelial and mesothelial cells (Song et al. 2009).

Entry of engineered NPs into the brain may be possible via several pathways. One of the well-studied pathways is the nose-to-brain transport through the olfactory epithelium. Olfactory sensory neurons (OSNs) reside in the olfactory epithelium and have processes in direct contact with the outside environment. Thus, NPs might be able to gain access to the brain by OSNs and its projections to the olfactory bulb and subsequently to other regions in the brain (Calderón-Garcidueñas et al. 2002; Oberdörster et al. 2004). Another possible nose-to-brain transport can occur through the trigeminal nerve, the largest of the cranial nerves. Afferent neurons from the trigeminal nerve pass directly through the nasal mucosa and enter the brainstem at the level of the pons (Mistry et al. 2009). For example, in rats, intranasal administration of the neurotrophic factor insulin-like growth factor-1 (IGF-1) resulted in the delivery of IGF-1 to both the olfactory bulb and brainstem areas (Thorne et al. 2004). In addition, NPs can be synthetically engineered to bypass the blood–brain barrier. One of the cardinal features of the blood–brain barrier is the tight junction that is formed between endothelial cells, restricting the passage of xenobiotics into the central nervous system (CNS). However, depending on their size and chemical composition, engineered NPs may bypass the BBB and gain direct entry into the brain (Lockman et al. 2004).

In the brain, microglia are immuno-competent cells and represent approximately 5–10% of the glial cell population. As the sentinels of the CNS, microglia are the first cells to respond to disruption of brain homeostasis and entry of foreign particles or infectious agents. Once activated, microglia can undergo morphological and functional changes, including proliferation, migration to the site of injury, and phagocytosis of cellular debris. Activated microglia can also generate reactive oxygen species (ROS) and reactive nitrogen species (RNS) and elicit an inflammatory response (Streit et al. 1999, 2005; Tambuyzer et al. 2009). Thus, microglia are the brain cells most likely to respond to the entry of NPs, and several studies have suggested this possibility (Kircher et al. 2003; Ribot et al. 2007; Voisin et al. 2007). Engineered NPs have been shown to trigger oxidative stress *in vitro*; because the brain is highly vulnerable to oxidative stress, increased ROS production can lead to neurodegeneration (Block et al. 2007; Long et al. 2007). One study showed that nanosize titanium dioxide, commonly used in skincare products, stimulates the production of ROS in immortalized brain microglia and damages neurons *in vitro* (Long et al. 2007). Other studies suggest the plausible role of ultrafine particles found in air pollution in neuroinflammation and in the induction of pathology associated with Alzheimer’s disease (Calderón-Garcidueñas et al. 2002, 2004).

Silica is a major component of sand and glass, and it has been used in the synthesis of NPs. Silica NPs (SiNPs) are generally deemed nontoxic and are inexpensive and easy to produce. Functional groups can also be added to the surface, making them appealing for designs for different applications (Roy et al. 2005; Wang et al. 2008). SiNPs are being formulated for potential drug delivery and for imaging and diagnostic applications in the CNS because they are considered to be more biocompatible than are other imaging NPs, such as quantum dots, which may contain toxic metals such as cadmium and mercury (Bharali et al. 2005; Liu et al. 2008; Manzoor et al. 2009; Roy et al. 2005; Su et al. 2009). Studies have proposed microglia as a vehicle for the delivery of NPs to tumors for imaging or drug delivery (Ribot et al. 2007; Voisin et al. 2007).

We synthesized the SiNPs used in this study using the Stöber method, which is known to generate amorphous SiNPs of a controlled size (Stöber et al. 1968). In addition, the SiNPs were embedded with a fluorescent dansylamide dye, allowing them to be tracked in cells. The physicochemical properties of these particular SiNPs have been previously characterized (Nyland et al. 2009). Incorporation of dansylamide dye molecules inside the silica matrix protects the dye from the surrounding environment and provides signal enhancement due to an increase in the number of dye molecules per NP without dye aggregations (Ow et al. 2005; Sokolov and Naik 2008). Energy-dispersive X-ray spectroscopy showed that these dansylamide-embedded SiNPs are not contaminated with other metals (Nyland et al. 2009). Another primary concern of testing the toxicity of NPs is the concept of NP aggregation, which can result in misleading information. Thus, we tested these SiNPs for stability and for clumping in cell culture media using scanning electron microscopy (SEM) and dynamic light scattering. These SiNPs were stable in cell culture media for approximately 1 week, and no clumping or aggregation was observed (Nyland et al. 2009; Thomassen et al. 2010). The goal of the present study was to determine whether these fluorescent SiNPs can be taken up by primary microglia from rat brain and to determine whether they disrupt microglial function.

## Materials and Methods

### Synthesis and characterization of SiNPs

The NPs used in this study were formed in an emulsion where 0.5 g of the hydrophobic phase, high-molecular-weight poly(dimethylsiloxane) (Scientific Polymer Products, Ontario, NY), was dissolved in 2 mL of the sol-gel precursor tetraethyl orthosilicate (Aldrich, Milwaukee, WI) and 70 mL ethanol (Aldrich), the hydrophilic phase. Then 0.05 g dansyl-aminopropyltriethoxysilane (dansylamide dye; Gelest, Morrisville, PA) was added to the solution and ultimately became covalently bonded to the cross-linked silica network. Control experiments established that this networking was essential for retention of the dye inside the particles. After adding 10 mL ammonium hydroxide (NH_4_OH) to initiate the polymerization process, 0.4 g Dow 190 surfactant (silicone ethylene oxide/propylene oxide copolymer; Dow Corning, Midland, MI) was added. The reaction was allowed to continue for 12 hr at room temperature. After the reaction was completed, NPs were washed with ethanol and deionized water to remove surfactant and then centrifuged five times. The NPs were finally resuspended in 100% ethanol. The final particle concentration was determined by averaging the number of counts in the field of view in 20 SEM images of a monolayer of 3.5 mg/mL NPs. The size, shape, and concentration of NPs (expressed as NPs per milliliter ethanol) were examined under SEM (JEOL 6700F) and an energy-dispersive X-ray spectroscopy microanalysis system (JEOL USA, Peabody, MA). Fluorescence of the dye-doped NPs was measured with a Hitachi F-4500 spectrophotometer (Hitachi, Schaumberg, IL). The fluorescence emission of the dansylamide-doped NPs was at 460–480 nm, a wavelength detectable using fluorescence microscopy.

**Figure 1:**
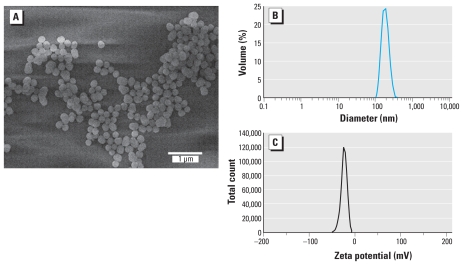
The physicochemical properties of the dansylamide-embedded SiNPs. (*A*) SEM image of the SiNPs (diameters range from approximately 150 to 200 nm; magnification, 19,000×). Dynamic light scattering data showing the size distribution by volume (~ 180 nm, polydispersity index = 0.038; *B*) and zeta potential distribution (−23 mV; *C*) of SiNPs.

The dansylamide-embedded SiNPs were also characterized before treatment. Figure 1A is an SEM image of the dansylamide-embedded SiNPs suspended in culture media. The NPs are spherical in shape and about 150–200 nm in diameter. These SiNPs do not aggregate in cell culture media, and the dansylamide dye molecules do not leak out as they are incorporated into the NP matrix. The size distribution and the surface charge of these SiNPs were measured by photon correlation spectroscopy using a Zetasizers 3000 (Malvern Instruments, Southborough, MA). The size distribution of these SiNPs (Figure 1B) indicates the absence of aggregation. The overall surface charge of these SiNPs, determined by their zeta-potential values, is negative (−23 mV; Figure 1C) because of the hydroxyl groups on their surface.

### Primary microglia cell culture

**Figure 2:**
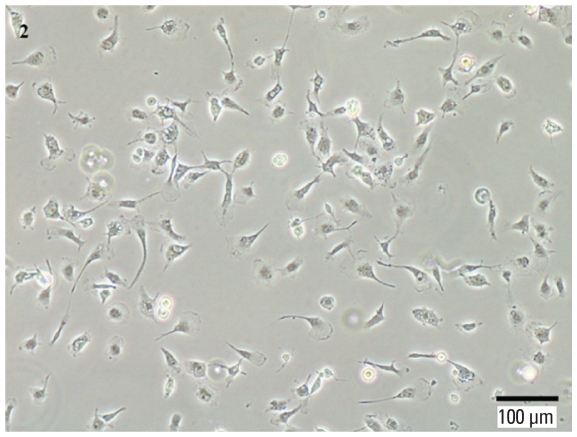
Light microscopy of primary rat microglia in culture. Primary rat microglia were extracted from mixed glial cultures and plated for 16–20 hr before use. Microglia appear healthy, with processes and a ramified morphology.

Primary mixed glial cell cultures were prepared using a modified version of the glial culture technique as previously described (Giulian and Baker 1986). Briefly, 9–10 brains from postnatal day 1–3 Sprague-Dawley rat pups (Harlan, Indianapolis, IN) were dissected, and the meninges were carefully removed. Brain tissue was dissociated by trypsination (0.25% trypsin at 37°C for 30 min), trituration, and filtration through 40-μm cell strainers. Cells were centrifuged at 2,000 × *g* for 10 min, resuspended, and plated onto 75-cm^2^ poly-l-lysine–coated culture flasks in Dulbecco’s modified Eagle medium (DMEM)/F12 (Invitrogen, Carlsbad, CA) containing 10% heat-inactivated fetal bovine serum (FBS; Hyclone, Logan, UT), and 100 U penicillin/100 μg streptomycin (Invitrogen). Cultures were maintained at 37°C in a humidified chamber of 95% air/5% CO_2_ for 12–14 days, when the glial cultures reached confluency. Microglia were then separated from the glial cultures by shaking the flasks for 2 hr at 200 rpm at 37°C and collected as floating cells in the media. After centrifugation (2,000 × *g* for 10 min), cell viability was determined by trypan blue exclusion, and cells were plated on 96-well plates, 6-well plates, or 60-mm dishes, depending on the assay being tested. Nonadherent cells were removed 20 min after plating by changing the culture medium to DMEM/F12 containing 2% FBS. Adherent cells were incubated overnight before being used for experiments. More than 95% of the adherent cells were positive for microglia-specific marker Mac-1 (Chemicon, Billerica, MA) as determined by immunostaining. Figure 2 shows an image of primary microglia in culture prior to treatment. The animals used for this study were treated humanely and with regard for alleviation of suffering. All the animal studies were reviewed and approved by the Johns Hopkins University Animal Care and Use Committee.

### Experimental conditions for SiNP treatments

We used a series of SiNP concentrations to test possible effects on primary microglia. The SiNP stock solution (concentrations ranging from 4 × 10^10^ NPs/mL to 7 × 10^10^ NPs/mL) was diluted in 100% ethanol to the appropriate concentrations (shown in Table 1), and the NPs were added to DMEM/F12 medium containing 2% FBS; ethanol content in the solutions was < 1%. Microglia were exposed to SiNPs for 24 hr before being assayed. In some experiments, bacterial lipopolysaccharide (LPS; Sigma-Aldrich, St. Louis, MO) was used as a positive control to assess microglial function. Table 1 shows the conversions of the NPs/volume concentrations used in this study to mass/volume. The concentrations we used were similar to or less than the concentrations used in previously published *in vivo* NP studies (He et al. 2008; Lockman et al. 2004).

### Cell viability

Cell viability was determined using Promega CellTiter 96 AQ_ueous_ Non-Radioactive Cell Proliferation Assay {MTS [3-(4, 5-dimethylthiazol-2-yl)-5-(3 carboxymethoxyphenyl)-2-(4-sulfophenyl)-2H-tetrazolium, inner salt]; Promega, Madison, WI} according to the manufacturer’s instructions. Briefly, 30,000 microglia/well were plated onto 96-well plates. Four hours before the end of the exposure, MTS was added, and at the end of exposure, the plate was read at 490 nm.

### Measurement of ROS

We measured ROS using the 2′,7′-dichlorohydrofluorescein diacetate (H_2_DCFDA) assay (Molecular Probes, Eugene, OR). Nonfluorescent H2DCFDA readily crosses the cell membranes and gets deacetylated to H_2_DCF by intracellular esterases. After oxidation by ROS, H_2_DCF is converted to the highly fluorescent DCF, which is detectable using a fluorescent plate reader. A 2.5-mM stock solution of DCF-DA was first made in dimethyl sulfoxide (DMSO; Sigma-Aldrich) and then diluted in Hank’s balanced salt solution (HBSS) so that the percentage DMSO in solution was < 1%. A total of 30,000 microglia/well was plated onto a 96-well plate. After 24-hr exposure, culture medium was removed and the cells were washed in HBSS. Cells were incubated for 30 min at 37°C in HBSS containing 10 μM DCF-DA. After incubation, medium was aspirated and the cells were washed twice with HBSS. The plate was read at 485 nm excitation and 530 nm emission.

### Measurement of RNS

We measured RNS using the 4-amino-5-methylamino-2′,7′-difluorofluorescein (DAF-FM) assay (Invitrogen). DAF-FM readily crosses the cell membranes and is deacetylated by intracellular esterases to 4,5-diaminofluorescein (DAF-2). DAF-2 remains essentially nonfluorescent until it reacts with the nitrosonium cation (produced by spontaneous oxidation of nitric oxide) to form a fluorescent benzotriazole, which is detectable using a fluorescent plate reader. A 5-mM stock of DAF-FM was first made in DMSO and then diluted in HBSS so that the percentage DMSO in solution was < 1%. We plated 30,000 microglia/well onto a 96-well plate. After 24-hr exposure, culture medium was removed and the cells were washed in HBSS. Cells were incubated for 30 min at 37°C in HBSS containing 1 μM DAF-FM. After incubation, medium was aspirated and the cells were washed twice with HBSS. The plate was read at 495 nm excitation and 515 nm emission.

### Phagocytosis

We measured microglial phagocytosis using fluorescent polystyrene microbeads (1 μm diameter; Molecular Probes). The emission wavelength for these beads is 598 nm, which does not overlap with the fluorescence of the SiNPs. A stock solution of 1 × 10^10^ beads/mL was diluted in HBSS to generate a solution of 50–100 fluorescent polystyrene beads per cell. We then plated 30,000 microglia/well onto a 96-well plate. After 24-hr exposure, medium was aspirated, and cells were washed and incubated for 30 min at 37°C in HBSS containing the fluorescent polystyrene beads. After incubation and washing, the plate was read at 570 nm excitation and 598 nm emission.

### SiNP uptake using fluorescence confocal microscopy and transmission electron microscopy (TEM)

We plated 250,000 primary microglia/well onto 6-well plates with a glass coverslip per well. After 24-hr exposure, microglial cells were fixed with 4% paraformaldehyde (15 min at room temperature) followed by 0.2% Triton (10 min at room temperature). The cells were then blocked in phosphate-buffered saline (PBS) containing 10% normal goat serum for 1 hr at room temperature before being incubated with mouse anti-Mac-1 primary antibody (1:100; Chemicon) overnight at 4°C. After three washes in PBS, the cells were incubated with goat anti-mouse Alexa 594 secondary antibody (1:500; Invitrogen). After three final washes in PBS, the cells were mounted with Prolong with DAPI (4,6-diamidino-2-phenylindole; Invitrogen) to counterstain for the nuclei. Fluorescence confocal images were taken using Zeiss LSM510-Meta (Carl Zeiss Microimaging Inc., Thornwood, NY) at the Johns Hopkins University (JHU) School of Medicine Microscope Facility.

For TEM, we plated 250,000 primary microglia/well per treatment onto 35-mm dishes. After 24-hr exposure, cells were washed and fixed in 2% glutaldehyde. The samples were then sent to the JHU School of Medicine Microscope Facility for electron microscopy preparation. TEM images were taken using a Hitachi 7600 TEM.

### Cytokine production profiling using Luminex (Luminex Corp., Austin, TX)

For profiling of cytokine production, we plated 30,000 primary microglia/well onto a 96-well plate. After 24-hr exposure, 50 μL of the culture medium was collected to be assayed for 10 different cytokines [granulocyte/macrophage colony–stimulating factor, interleukin (IL)-1α, IL-1β, IL-2, IL-4, IL-6, IL-10, IL-12, interferon-γ, and tumor necrosis factor (TNF-α)] using the Cytokine Rat 10-Plex Panel (Invitrogen) according to the manufacturer’s instructions.

### Changes in gene expression using quantitative real-time polymerase chain reaction (qRT-PCR)

We plated 500,000 primary microglia per treatment onto 60-mm dishes. After 24-hr exposure, total RNA was isolated from each plate using the Qiagen RNeasy Mini Kit (Qiagen, Valencia, CA) according to the manufacturer’s instructions. The quality of RNA was tested using an Agilent Bioanalyzer (Quantum Analytics, Inc., Foster City, CA). We used 50 ng total RNA for reverse transcription using the Sensiscript RT Kit (Qiagen) according to manufacturer’s instructions. The final cDNA product was diluted to a final volume of 100 μL, and 5 μL of cDNA per gene of interest was used for qRT-PCR, which was performed using the Applied Biosystems Real-Time PCR 7000 Sequence Detection System with Taqman Universal PCR Master Mix and rat *TNF*-α [GenBank accession no. NM_012675.2 (http://www.ncbi.nlm.nih.gov/Genbank/)] and rat cyclooxygenase-2 (*COX-2*; GenBank accession no. NM_017232.3) Taqman primers (all from Applied Biosystems, Foster City, CA). β-Actin was used as the internal control for these experiments. qRT-PCR analysis was performed using the ΔΔCt method.

### Statistical analysis

Values are expressed as mean ± SE. Each group consisted of three or four independent trials for each concentration studied. We used a one-way analysis of variance to determine treatment effect followed by Fisher’s post hoc analysis.

## Results

### Uptake of SiNP by primary microglia

**Figure 3:**
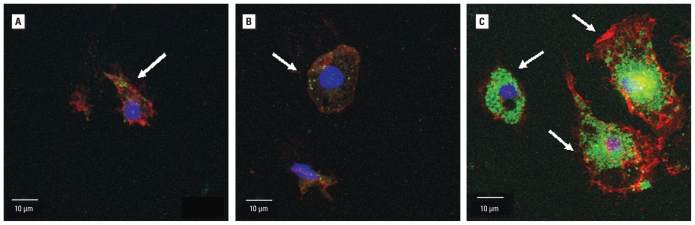
Uptake of SiNPs by primary microglia detected by fluorescence confocal microscopy, showing intracellular accumulation of SiNPs at three concentrations tested: 4 NPs/μL (*A*), 400 NPs/μL (*B*), and 40,000 NPs/μL (*C*). Arrows indicate cells that have accumulated SiNPs. It appears that microglia became hypertrophic with increased SiNP concentrations, based on the expansion of their cytoplasm. SiNPs are shown in green; Mac-1 (red) is a microglia-specific marker, and DAPI (blue) is a nuclei-specific marker.

**Figure 4:**
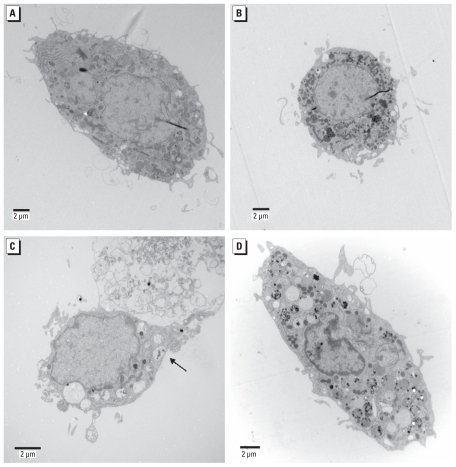
Uptake of SiNPs by primary microglia revealed by TEM. (*A*) Image of a normal microglia (magnification, 5,000×). (*B*) Microglia exposed to 4 NPs/μL; SiNPs were dispersed throughout the cytoplasm (magnification, 5,000×). (*C*) Microglia exposed to 400 NPs/μL; there was uptake of SiNPs into what it appears to be phagocytic vacuoles (arrow; magnification, 8,000×). (*D*) Microglia exposed to the highest concentration tested (40,000 NPs/μL); numerous phagocytic vacuoles contained SiNPs (magnification, 6,000×).

**Figure 5:**
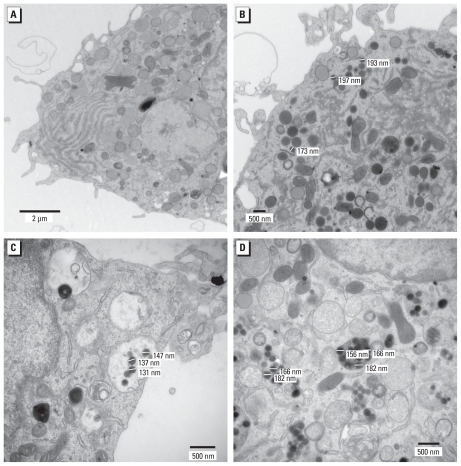
TEM images showing the diameters of the SiNPs inside the microglia. (*A*) Normal microglia with no SiNPs (magnification, 12,000×). (*B*–*D*) Higher-magnification TEM images of microglia exposed to 4 NPs/μL (*B*; magnification, 12,000×), 400 NPs/μL (*C*; magnification, 30,000×), and 40,000 NPs/μL (*D*; magnification, 25,000×). Diameters of SiNPs inside the microglia ranged from 131 nm to 197 nm.

We exposed primary microglia to various concentrations of SiNPs for 24 hr. Confocal fluorescent microscopy showed the internalization of SiNPs by microglia at all concentrations tested (Figure 3). At the highest concentration, 40,000 NPs/μL (equivalent to 7.28 μg/mL), nearly every microglia showed uptake of SiNPs. At this concentration, primary microglia looked hypertrophic compared with lower concentrations (Figure 3C). We also detected uptake of SiNPs by TEM. At 4 NPs/μL (7.28 × 10^−4^ μg/mL), SiNPs were present within the cytoplasm but did not appear to accumulate in phagocytic vacuoles (Figures 4B, 5B). In contrast, at 400 NPs/μL (7.28 × 10^−2^ μg/mL; Figures 4C, 5C) and 40,000 NPs/μL (7.28 μg/mL; Figures 4D, 5D), SiNPs were present in what appear to be phagocytic vacuoles. Analysis of the diameter of SiNPs in the cytoplasm and in phagocytic vacuoles of microglia (Figure 5B–D) is consistent with the known distribution of the diameter for most of the SiNPs, which is approximately 150–200 nm (Figure 1B).

### Cell viability and phagocytosis

**Figure 6:**
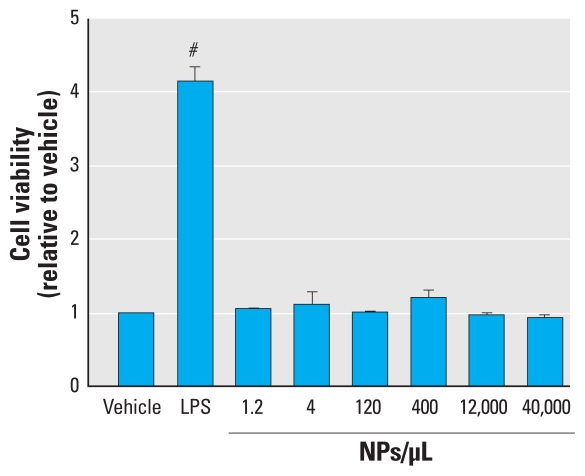
Effects of SiNPs on microglial cell viability. Data are normalized to vehicle and expressed as mean ± SE of three independent trials. ^#^*p* < 0.05 compared with all treatments.

We examined whether SiNPs are cytotoxic to microglia, by measuring cell viability using the MTS assay. We used LPS, a potent inflammagen, as a positive control because it is a well-known activator of microglia (Facchinetti et al. 2003; Minghetti and Levi 1995). After a 24-hr exposure to SiNPs, we observed no significant difference in cell viability at any of the SiNP concentrations tested relative to the vehicle control (Figure 6). However, LPS generated a positive response compared with vehicle and all the SiNP concentrations tested (*F*_7,19_ = 32.67; *p* = 0.0001), which may be related to an effect of LPS on microglial proliferation.

**Figure 7:**
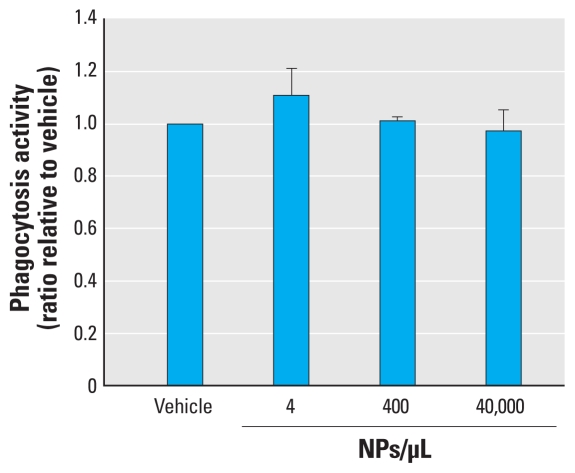
Effects of SiNPs on microglial phagocytosis, determined using fluorescent polystyrene microspheres as a marker for phagocytosis. Data are normalized to vehicle and expressed as mean ± SE of three independent trials.

To determine whether SiNPs can alter phagocytic activity, we exposed primary microglia to SiNPs for 24 hr before the addition of fluorescent polystyrene microspheres, which are commonly used to assess phagocytic activity. The microspheres are detected at a different wavelength (emission at 598 nm) than the SiNPs (emission at 460–480 nm), so SiNP fluorescence does not interfere with this phagocytic activity assay. Figure 7 shows that SiNPs did not produce a significant effect on microglial phagocytic activity (*F*_3,8_ = 0.775; *p* = 0.540). We also tested phagocytosis at an earlier time point of SiNP exposure (i.e., 30 min), and did not observe a difference in phagocytic activity (data not shown). In summary, SiNPs at the concentrations tested were not cytotoxic to primary microglia, nor did they alter phagocytosis.

### Production of ROS and RNS

**Figure 8:**
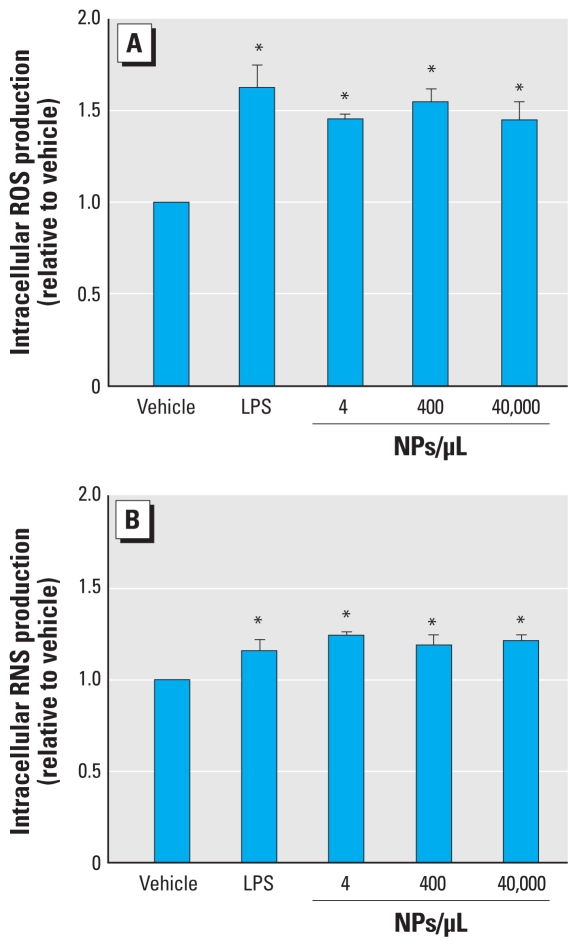
Effects of SiNPs on microglial production of intracellular ROS (*A*) and RNS (*B*). Data are normalized to vehicle and expressed as mean ± SE of three independent trials. *p < 0.05 compared with vehicle.

Although cell viability was not altered by SiNPs, we were interested in determining whether SiNPs could induce the production of intracellular ROS and RNS. It is known that, upon activation, microglia are capable of generating both ROS and RNS. After 24 hr exposure to SiNPs, we observed a consistent and significant increase in intracellular production of ROS (*F*_4,11_ = 8.47; *p* = 0.0023; Figure 8A) and RNS (*F*_4,11_ = 9.85; *p* = 0.0012; Figure 8B). These increased levels of intracellular ROS and RNS were present even at the lowest concentration tested, and were similar to the level produced by 100 ng/mL LPS (Figure 8).

### Gene expression of proinflammatory markers

**Figure 9:**
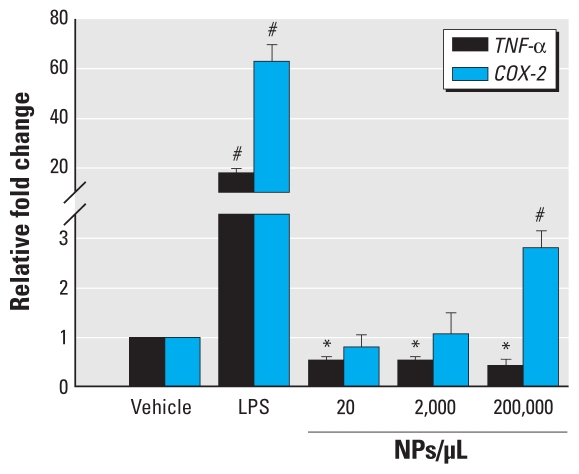
Effects of SiNPs on inflammatory gene expression of *TNF-*α and *COX-2* measured by qRT-PCR. Data are normalized to vehicle and expressed as mean ± SE of three independent trials. *p < 0.05 compared with vehicle and 100 ng/mL LPS of three independent trials. ^#^*p* < 0.05 compared with all treatments.

To determine if exposure to SiNPs can induce an inflammatory response in primary microglia, we measured the levels of the proinflammatory genes *TNF-*α and *COX-2* using qRT-PCR. After 24 hr of SiNP exposure, we observed a significant decrease in *TNF-*α gene expression at all concentrations tested (*F*_4,12_ = 79.27; *p* = 0.0001) and a significant increase in *COX-2* gene expression at the highest concentration of 200,000 NPs/μL (36.4 μg/mL of SiNPs; (*F*_4,10_ = 43.30; *p* = 0.0001) (Figure 9).

### Cytokine release

**Figure 10:**
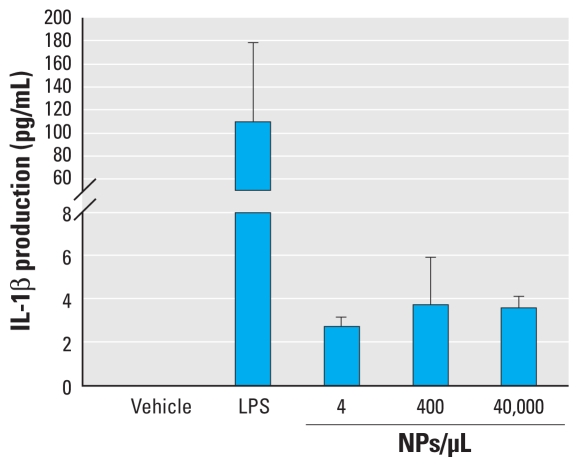
Effects of SiNPs on release of the cytokine IL-1β. Data are expressed as mean ± SE amount of IL-1β released from three independent trials.

We examined the effect of SiNPs on the release of inflammatory cytokines, using Luminex technology, which can simultaneously measure 10 different cytokines. Under the conditions used, we measured low but detectable levels of IL-1β after SiNP exposure. In contrast, IL-1β was not detectable in the culture medium from vehicle-exposed microglia. Microglia treated with 100 ng/mL LPS served as a positive control, and this treatment resulted in a robust increase in IL-1β release (Figure 10).

## Discussion

In this article we provide *in vitro* evidence for the effects of SiNPs on primary microglia from rat brain. Using both TEM and fluorescence confocal microscopy, we found that primary microglia can take up SiNPs when exposed to very low concentrations (as low as 4 NPs/μL, or 7.28 × 10^−4^ μg/mL). Although these SiNPs do not exert acute cytotoxicity at the concentrations tested or alter phagocytic activity of microglia, SiNPs did increase intracellular ROS and RNS levels. Previous studies have shown that intracellular ROS acts as a signal that stimulates the production of inflammatory markers (Pei et al. 2007; Wang et al. 2004). This is consistent with our observation that microglia exposed to SiNPs released detectable levels of IL-1β. We also observed a consistent decrease in *TNF-*α gene expression at all SiNP concentrations and a significant increase in *COX-2* gene expression at the highest concentration. We do not yet understand the reason for the decrease in *TNF-*α gene expression. A change in COX-2 activity may be involved. Previous studies have shown that enhanced COX-2 activity and subsequent increase in prostaglandins such as PGE_2_ (prostaglandin E_2_) has a negative feedback in TNF-α (Aloisi et al. 1999; Levi et al. 1998; Zhang and Rivest 2001). PGE_2_ might exert this effect by increasing the level of intracellular cAMP because the effect of PGE_2_ on down-regulating TNF-α was mimicked by cAMP-elevating agents (Minghetti et al. 1997). However, this is only speculative, and future studies are needed to examine this possibility.

An interesting observation from this study is that at the lowest concentration (4 NPs/μL; 7.28 × 10^−4^ μg/mL), the SiNPs did not appear to be localized in phagocytic vacuoles but were dispersed throughout the cytoplasm. In contrast, at higher concentrations, SiNPs appeared to be engulfed in phagocytic vacuoles. This suggests different mechanisms of SiNP uptake based on SiNP concentration. Future studies will examine the effects of concentration, size, and surface modification of SiNPs on primary microglial uptake and function, because smaller SiNPs with a higher surface area can elicit a greater dose-dependent cytotoxicity than larger NPs (Lin et al. 2006; Napierska et al. 2009; Shvedova et al. 2007).

This study provides important insights on possible adverse effects on primary microglia exposed to SiNPs *in vitro*. The results can guide experimental animal studies in which SiNPs are administered *in vivo*. This is an important next step because reports have demonstrated the use of SiNPs for drug delivery into the CNS without assessing potential toxic effects. Our present results showed the effects of only 24-hr exposures to SiNPs. However, if SiNPs are not readily degradable or eliminated from the CNS, then it is probable that SiNPs would accumulate in the brain, leading to an exacerbation of the microglial responses observed in this study.

## Conclusion

This is the first report of effects of SiNPs on primary microglia. The main effect observed is increased production of intracellular ROS and RNS, altered *TNF-*α and *COX-2* gene expression, and a small but detectable release of IL-1β. Other studies have suggested that microglia can internalize SiNPs and be used as a vehicle for gene therapy and as a contrast agent for glioma treatment (Kircher et al. 2003; Ribot et al. 2007; Voisin et al. 2007). Although this approach may advance the field of nanomedicine, little is known about the possible adverse effects of SiNPs once they have been internalized by microglia in the brain parenchyma. Our study suggests that at concentrations as low as 4 NPs/μL (7.28 × 10^−4^ μg/mL), there is a significant increase of ROS and RNS. Because NPs may gain entry into the brain via multiple pathways, it is possible that microglia may be affected in both supratentorial and infratentorial regions of the brain. This could cause deleterious effects, because the continuous production of ROS by microglia is capable of damaging neurons in their vicinity. Moreover, SiNP-induced microglial dysfunction can lead to their inability to maintain neuronal health.

## Figures and Tables

**Table 1:** Conversion of concentrations used (nanoparticles/volume) to mass/volume

NPs/μL	Mass/volume (μg/mL)
1.2	2.18 × 10^−4^
4	7.28 × 10^−4^
20	3.64 × 10^−3^
120	0.0218
400	0.0728
2,000	0.364
12,000	2.18
40,000	7.28
200,000	36.4
